# Pancreatic Cancer Diagnosis and Management: Has the Time Come to Prick the Bubble?

**DOI:** 10.3389/fendo.2018.00779

**Published:** 2019-01-08

**Authors:** Pedro Moutinho-Ribeiro, Guilherme Macedo, Sónia A. Melo

**Affiliations:** ^1^Department of Gastroenterology, Centro Hospitalar São João, Porto, Portugal; ^2^Faculty of Medicine of the University of Porto, Porto, Portugal; ^3^Institute for Research Innovation in Health (i3S), Porto, Portugal; ^4^Institute of Pathology and Molecular Immunology of the University of Porto, Porto, Portugal

**Keywords:** pancreatic cancer, early diagnosis, liquid biopsy, biomarkers, exosomes

## Abstract

Pancreatic cancer (PC) is associated with poor prognosis and very dismal survival rates. The most effective possibility of cure is tumor resection, which is only possible in about 15% of patients diagnosed at early stages of disease progression. Recent whole-genome sequencing studies pointed genetic alterations in 12 core signaling pathways in PC. These observations hint at the possibility that the initial mutation in PC might appear nearly 20 years before any symptoms occur, suggesting that a large window of opportunity may exist for early detection. Biomarkers with the potential to identify pre-neoplastic disease or very early stages of cancer are of great promise to improve patient survival. The concept of liquid biopsy refers to a minimally invasive sampling and analysis of liquid biomarkers that can be isolated from body fluids, primarily blood, urine and saliva. A myriad of circulating molecules may be useful as tumor markers, including cell-free DNA (cfDNA), cell-free RNA (cfRNA), circulating tumor cells (CTC), circulating tumor proteins, and extracellular vesicles, more specifically exosomes. In this review, we discuss with more detail the potential role of exosomes in several aspects related to PC, from initiation to tumor progression and its applicability in early detection and treatment. Exosomes are small circulating extracellular vesicles of 50–150 nm in diameter released from the plasma membrane by almost all cells and exhibit some advantages over other biomarkers. Exosomes are central players of intercellular communication and they have been implicated in a series of biological process, including tumorigenesis, migration and metastasis. Several exosomal microRNAs and proteins have been observed to distinguish PC from benign pancreatic diseases and healthy controls. Besides their possible role in diagnosis, understanding exosomes functions in cancer has clarified the importance of microenvironment in PC progression as well as its influence in proliferation, metastasis and resistance to chemotherapy. Increasing knowledge on cancer exosomes provides valuable insights on new therapeutic targets and can potentially open new strategies to treat this disease. Continuous research is needed to ascertain the reliability of using exosomes and their content as potential biomarkers, so that, hopefully, in the near future, they will provide the opportunity for early diagnosis, treatment intervention and increase survival of PC patients.

## Introduction

Pancreatic ductal adenocarcinoma (PDAC), the most frequent type of pancreatic cancer (PC), is associated with poor prognosis, with 53,670 new cases and 43,090 estimated deaths in 2017 (1). It represents around 3% of the new cancer cases each year, but it is the fourth most common cause of cancer mortality (2). It is expected to become the second cause of death by cancer by 2020 in the USA (2). On the contrary to the death rates for many other cancer, such as lung, colorectal, breast, and prostate, the death rate for PDAC patients has increased (3) as well as its incidence, that has raised in about 30% (2).

Pancreatic cancer is typically asymptomatic in its first stages of development, and as a consequence of the late diagnosis, this disease presents a very low survival rate. Combining all stages, the overall 5-year survival rate is 5% (4). Tumor resection is the only possibility of cure, but recurrence often happens and, therefore, the 5-year survival rate in resected patients is only up to 25% (1). Its unique tumor biology contributes to early recurrence, metastasis, and a subpar response to conventional therapies (5).

Since lifetime risk of PC in general population is low (1.3%), a population-based screening is not recommended (6). Meanwhile, individuals with family history or genetic predisposition have been identified as risk groups. According to the International Cancer of the Pancreas Screening Consortium (CAPS) it has been proposed that these individuals at higher risk for PC should be considered for screening (6, 7). The referred Consortium defined eligible individuals to be those with more than 5% of lifetime risk or with a 5-fold increased relative risk for PC (6, 7). Nonetheless, this group of patients represents only 10% of the total spectrum of PC, the other 90% of cases being sporadic (8).

## Pancreatic Cancer Precursor Lesions: a Window for Opportunity

Recent studies based on whole-genome sequencing indicated 12 important signaling pathways altered in PC. It was also suggested that the initial mutation occurred nearly 20 years before the first symptoms. This evidence has offered a time frame for pancreatic carcinogenesis, suggesting that a large window of opportunity may exist for early detection, which could improve the prognosis of this lethal disease (9, 10).

In recent years, a myriad of biomarkers have been investigated with specificity for PC detection (8). The ideal biomarker should be able to detect the disease in its early stages, when patients are still amenable for a curative treatment, or even in a more favorable scenario, its premalignant precursor lesions. Pancreatic cancer precursor lesions are intraductal papillary mucinous neoplasms (IPMNs), the mucinous cystic neoplasms (MCNs), and the pancreatic intraepithelial neoplasias (PanINs), the first two being macroscopic cystic alterations and the last one representing microscopic non-invasive epithelial proliferations within the pancreatic ducts (5). Intraductal papillary mucinous neoplasms (IPMNs) are papillary proliferations inside the pancreatic ducts that usually secrete thick mucus, leading to its focal dilation. They represent up to 10% of the neoplasms of the pancreas, and the ones harboring high-grade dysplasia carry an important risk of malignant transformation (11, 12). Depending on the extent of ductal involvement, three subtypes can be recognized: the main-duct intraductal papillary mucinous neoplasm (MD-IPMN), the branch duct intraductal papillary mucinous neoplasm (BD-IPMN), and the mixed type. Malignant transformation is more frequent in main duct and mixed types IPMNs, while BD-IPMNs are considered indolent lesions (11, 12).

In relation to cytoarchitectural and immunophenotypic features, four histological subtypes of IPMNs are considered: the gastric type (49–63%), the intestinal type (18–36%), the pancreaticobiliary type (7–18%), and the oncocytic (1–8%) type (11, 13). Main-duct intraductal papillary mucinous neoplasm (MD-IPMNs) are more frequently of the intestinal type, a combination that carries the highest risk of invasive transformation, usually giving rise to a colloid type carcinoma. On the other hand, the branch duct intraductal papillary mucinous neoplasm (BD-IPMN) are predominantly of the gastric type, which are characterized by an insignificant risk of malignant transformation. The pancreatobiliary type usually harbors high-grade dysplasia and is considered the aggressive evolution of the gastric type. Finally, the rarer oncocytic type presents with cytological atypia, frequently with high-grade dysplasia (14).

MCNs, unlike IPMNs, are neoplasms that develop in the pancreatic parenchyma, without involvement of the ductal system. Characteristically they are seen in women, rarely occurring in men, with a preferential location in the body and tail of the gland (15).

An ovarian-type stroma is an essential feature for the diagnosis of MCNs that clearly separates them from the much more frequent IPMNs. Depending on the grade of cytologic atypia in epithelial lining (low, intermediate or high-grade dysplasia), which has been associated with point mutations in KRAS and p53 genes, mucinous cystic neoplasms (MCNs) may exhibit different risks of malignant potential (15–17). Interestingly, the observation that in invasive MCNs, the inactivation of SMAD4/DPC4 suppressor gene complex occurs only in the epithelial lining but not at the stroma level, suggests that the typical ovarian-type stroma of these lesions is not involved in the process of malignant transformation (18). The reported incidence of invasive carcinoma among MCNs is variable according to different series, ranging from 6 to 36% (15, 19, 20).

With the development and widespread of imaging modalities, the diagnosis of both of these macroscopic cystic PC precursor lesions is increasing. As the majority of them are asymptomatic and discovered incidentally, its proper management is not consensual and is a matter of debate by many International Societies worldwide. In fact, despite the availability of a significant number of guidelines and recommendations (21), there is still a lack of consensus in the decision of which one(s) to follow.

The vast majority of carcinomas originate from microscopic non-invasive epithelial proliferations within the pancreatic ducts, described as PanIN (22). These lesions are considered precursors in the stepwise progression from intraepithelial to invasive neoplasia. This morphological progression is accompanied by accumulation of genetic changes, in which activating KRAS mutations are thought to be the driving force (8, 12). PanIN lesions are characteristically asymptomatic and are composed of columnar to cuboidal cells with varying amounts of mucin and different degrees of cytological and architectural atypia (23). These lesions are classified into 3 grades: PanIN-1A (flat) and PanIN-1B (papillary) are low-grade lesions with minimal cytological and architectural atypia, while high-grade PanINs (PanIN-3), also described as “carcinoma *in situ*,” are characterized by severe cytological and architectural atypia (23). In cadavers of patients over the age of 80 the prevalence of PanIN lesions is about 55%. Also, these lesions are very frequent in patients with concomitant PC and in familial PC kindreds. The major problem concerning these very frequent lesions is their identification and the evaluation of its malignant potential. Currently, there is no imaging technique capable of an accurate diagnosis of a PanIN lesion and many promising biomarkers are being investigated for this purpose.

## Conventional Diagnostic Tools are Insufficient for Early Detection

**Figure 1:**
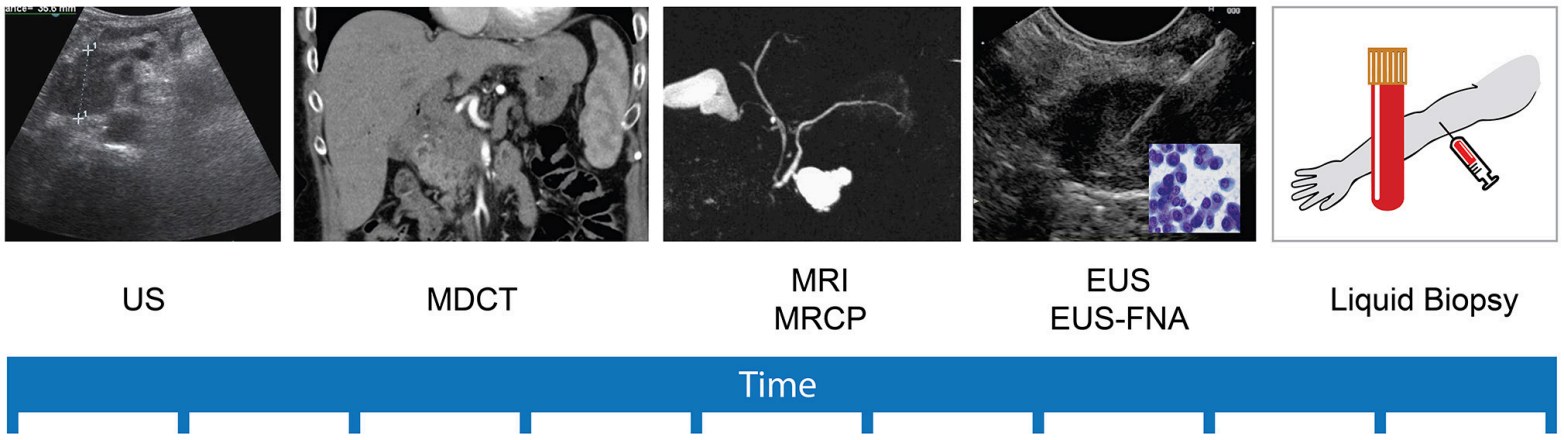
Timeline of development of pancreatic cancer diagnostic and staging modalities. Pancreatic cancer (PC) diagnosis and staging depends, substantially, on imaging modalities. Abdominal Ultrasonography (US) was the first to appear, but lacks sensitivity to detect small treatable lesions. Multidetector computed tomography (MDCT) is nowadays frequently used to detect and stage pancreatic masses, with good accuracy specially for 2 cm and larger lesions. Magnetic resonance imaging (MRI) and its variant magnetic resonance cholangiopancreatography (MRCP) improved the sensitivity for characterization small cystic lesions. More recently, endoscopic ultrasonography (EUS) with the possibility to perform fine needle aspiration (FNA) constitutes a prime modality for precise diagnosis and local staging of small (< 2 cm) solid and cystic pancreatic lesions. The innovative concept of liquid biopsy refers to a simple and painless collection of a body fluid sample (usually blood), in order to study proved and anticipated biomarkers with the potential to detect PC in its early non detectable stages and even the premalignant precursors.

Early diagnosis of PC is very challenging with the currently available methods (Figure 1) (24). Unlike colonoscopies for colorectal cancer and serum prostate-specific antigen (PSA) levels for prostate cancer, there is currently no standardized PC screening strategy, even for high-risk populations. Pancreatic cancer diagnosis and staging depends, substantially, on imaging modalities including ultrasonography (US) and endoscopic ultrasonography (EUS), multidetector computed tomography (MDCT), magnetic resonance imaging (MRI) and magnetic resonance cholangiopancreatography (MRCP), endoscopic retrograde cholangiopancreatography (ERCP), and positron emission tomography (PET) (25–30).

Nowadays, multidetector computed tomography (MDCT) is the imaging technique of choice for pancreatic diseases, especially in the setting of solid tumors, where it has high accuracy to detect and to stage pancreatic malignancies (31). However, its sensitivity may be suboptimal as it misses some target lesions in the PC screening context. Even when considering thin-section, triple-phase helical CT, the sensitivity to detect lesions smaller than 2 cm is only up to 80% (32). Moreover, ionizing radiation exposition is also an important drawback, precluding CT to be an ideal screening and/or surveillance imaging technique.

In high-risk individuals, magnetic resonance imaging (MRI) can be used as a non-invasive screening imaging test considering the possibility to scan the entire abdomen and pelvis, avoiding radiation exposure. Considering the MRCP, this technique offers, in a non-invasive way (in contrast to ERCP), the capacity to characterize the ductal anatomy of the pancreas and diagnose small cystic lesions such as IPMNs. Preliminary data from CAPS3 study, that included high-risk patients submitted to surgical resection, suggests that MRI/MRCP may be superior to CT particularly for detection of IPMNs (71 vs. 14%, *p* < 0.001) (33). Some MRI features have been recently added to this technique improving its diagnostic capacity. In fact, diffusion-weighted imaging (DWI), a technique based on the Brownian motion of water molecules in tissue (34), has brought functional aspects into conventional anatomic evaluation, allowing higher contrast resolution and the identification of very small PC lesions.

In diagnosing PC, the advantage of EUS over MDCT has been reported for more than 10 years (35, 36). Despite being an imaging technique operator-dependent, with inherent risks of invasiveness and sedation, EUS has progressively being considered as the most accurate tool to investigate pancreatic diseases. It is also a non-radiation technique that can offer high-resolution images and can accurately characterize solid and cystic lesions. Moreover, it can evaluate cystic wall features associated with increased risk of malignancy, namely mural nodules, and other focal thickenings. The data analysis from a screening program involving high-risk individuals that were submitted to surgical resection confirmed the superiority of EUS, as it was able to detect almost twice as many neoplastic lesions comparing to MRI/MRCP or CT (33). Also, past studies (37, 38) that used EUS as a screening tool showed its accuracy in detecting asymptomatic precancerous branch duct IPMNs, large PanINs, incidental pancreatic endocrine tumors and ductal adenocarcinomas. The chance to add some recent techniques such as EUS-guided elastography and contrast-enhanced imaging has expanded new and promising fields of investigation (37–40).

Considering EUS-guided fine-needle aspiration (EUS-FNA), when performed in suspected lesions, it shows diagnostic accuracy for malignancy of more than 85–90%. Apart from its role in the study of solid lesions, the investigation of pancreatic cystic lesions by EUS-FNA can also be very useful, allowing a cytological diagnosis of IPMN and MCN in up to 70% of the cases (41). “Cell-block preparation” and “core tissue sampling” are two developments of this technique (42), which might be useful not only in providing more material for histological evaluation, but also for recently developed ancillary diagnostic techniques, namely: microRNA profiling, KRAS mutation detection and chemo sensitivity testing (43–45).

Lately, confocal laser endomicroscopy has surged as a technological improvement to EUS with a particular interest in cystic lesions characterization. In this technique, a dedicated miniprobe is introduced through a 19-gauge needle previously inserted into the cystic lesion, allowing a real time direct visualization of the epithelial lining at a microscopic level, permitting the identification of suspicious architectural changes (46).

Nowadays, no biomarker exists with adequate sensitivity and specificity for routine clinical diagnosis or screening of PC (8). Carbohydrate antigen (CA) 19-9 and carcinoembryonic antigen (CEA) are the most commonly used blood-based tumor biomarkers in clinical practice. The only biomarker approved by the US FDA for monitoring the progression and therapeutic response of PC is CA 19-9, which is also the only one recommended by the NCCN guidelines for clinical management of PC patients (47). Nevertheless, it is not specific and can be found in normal pancreatic and biliary duct cells, as well as in gastric, endometrial, colonic, and salivary epithelia. In fact, depending on the series, its specificity ranges from 30 to 100% (48–50), so the extremely high rate of false positive results prohibits its routine use for diagnosis. Moreover, CA 19-9 sensitivity is also imperfect, ranging from 41 to 86% (48–50). Additionally, up to 15% of general population do not express Lewis antigen and, consequently, CA 19-9 levels cannot be measured (50–53). Another clinically relevant issue is the fact that only 65% of patients with resectable tumors have increased levels of CA 19-9. In relation to its ability to differentiate PC from chronic pancreatitis, this biomarker is also inadequate, as up to 40% of patients harboring this last condition exhibit CA 19-9 levels above the normal range (52, 54). Considering all these pitfalls, serum CA 19-9 is used primarily as a prognostic tool in monitoring patients for recurrence or managing those with late stage disease (52, 55).

Taking the aforementioned aspects in account, it is understandable that most patients present with advanced disease, with only up to 25% having resectable tumors (56–58). We should also have in mind that even if these 25% of patients are identified and properly treated under the current standards of care, their survival, at the best, is up to 24 months (4). So, the focus should be, on one side, in aiming to detect the “real” curable lesions, that is the premalignant and the very small malignant ones, and, on the other side, the development of better therapeutic options. For the early detection scenario, the conventional diagnostic tools are far from being competent. In fact, available non-biopsy tests (serum CA19-9) and imaging techniques lack the sensitivity and specificity necessary to detect tumors smaller than 1 cm in the context of a 1.5% lifetime risk disease (59–65).

In consequence, it is crucial to develop new and improved strategies which can overtake all the obstacles described and diagnose primary tumors that can be resectable at very early stage. The development of markers with high sensitivities and specificities for PC and for its precursor conditions should be a priority issue, with extreme importance mainly in the context of high-risk individuals (66).

## Liquid Biopsy, a Horizon for Early Detection and Survival Improvement

As mentioned before, given the typical late stage of disease at the time of presentation, when treatment is disappointing, PC remains one of the most dismal diseases worldwide, with incidence nearly parallel to mortality. There has been much effort invested in identifying accurate tumor markers, ideally present in the timeframe between cancer onset and invasion, to allow diagnosing PC in early curable stages, to ultimately improve patient's survival (67, 68). In this setting, the ideal biomarker should be easily detected with satisfactory sensitivity and specificity and should distinguish PC from other benign pancreatic lesions. In the context of early detection, the identification of preneoplastic conditions, such as PanINs, IPMNs and MCNs, is of great importance (69).

The concept of liquid biopsy refers to the analysis of biomarkers present in a sample of a body fluid collected through a simple and painless, minimally invasive technique. The body fluids mostly used for biomarker isolation are essentially blood, urine and saliva (70–72). A myriad of circulating molecules may be used as tumor markers, including cell-free DNA (cfDNA), cell-free RNA (cfRNA), circulating tumor cells (CTC), circulating tumor proteins, and extracellular vesicles, more specifically exosomes (66, 73). Blood is easily accessible and relatively stable, making serum an ideal specimen to explore potential biomarkers. However, biomarkers secreted into serum are extremely diluted and probably obscured by other more-abundant serum proteins (74). Technological advances in the last decade have provided more opportunities to discover circulating biomarkers based on “omics” analyses, including methods focused on proteins, nucleic acids, CTCs, and exosomes. Numerous proteins of low abundance can be analyzed by mass spectrometry-based approaches and proteomic technologies (24).

In recent years, based on the expression of transcriptional profiles and structural variations, different molecular subtypes of PC have been described thought genomic analyses (75–78). The early detection of mutant genes that identify PC and its subtypes is essential for an effective strategy for the management of the disease.

In PDAC, there are four major driver genes (one oncogene and three tumor suppressor genes) implicated in tumorigenesis (5). KRAS is the most frequently altered oncogene that encodes a GTPase which mediates downstream signaling from growth factor receptors; somatic mutations, clustered in specific hotspots (most in codon 12), are identified in more than 90% of PC (79). CDKN2A, by turn, is the most frequently altered tumor suppressor gene, with loss of function in more than 90% of tumors; it encodes an important cell-cycle regulator (79). TP53 is another tumor suppressor gene, with essential role in cellular response to stress, also exhibits frequent somatic mutations (79). Lastly, SMAD4, a tumor suppressor gene mediating downstream signaling of the transforming growth factor β (TGFβ) receptor is inactivated in about half of the PC cases (79).

Since Mandel and Metais, in 1948, (80) first described circulating free DNA in body fluids, an exponential interest in non-invasive technology for disease monitoring has been the focus of research in many centers worldwide.

Recently, due to the possibility to pair genomic tests with tests on CTCs, circulating tumor nucleic acids (ctNAs) and tumor-derived exosomes, liquid biopsies have gained increased value for clinical application (71, 81, 82).

During PC initiation and progression, many different genetic modifications take place, including genetic diversification, amplifications and homozygous deletions, an increase in duplicate chromosomal number, recapitulation of clonal expansion, clonal selection, driver mutations and losses of heterozygosity (10, 83–90).

Next-generation sequencing techniques provide deeper insight into somatic mutations and epigenetics analysis of the genome and broaden the characterization of circulating tumor DNA (ctDNA) and (cfRNA). With the development of cell tracking techniques and flow cytometry, it is now possible to capture and analyze CTCs and exosomes (24).

**Figure 2:**
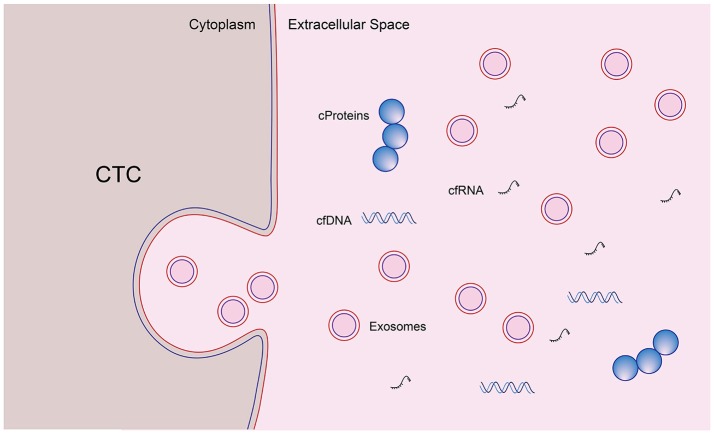
Current understanding of molecular biomarkers for pancreatic cancer. A myriad of circulating molecules may be used as tumor markers, including cell-free DNA (cfDNA), cell-free RNA (cfRNA), circulating tumor cells (CTC), circulating tumor proteins, and extracellular vesicles, more specifically exosomes. The characterization of these tumor biomarkers favors improvement of the knowledge of tumor heterogeneity and, most importantly, contributes to early detection, monitoring of disease progression and response to treatment. Moreover, some of these molecules, in the near future, can play a role as therapeutic targets, hopefully allowing the control of the disease during its early curable stages.

In this manner, the capabilities of liquid biopsy are enormous, allowing the characterization of tumor biomarkers in the same way tissue biopsy does, favoring improvement of the knowledge of tumor heterogeneity and, most importantly, contributing to early detection, monitoring of disease progression and response to treatment (Figure 2) (71, 82, 91–96).

## Exosomes: the Bubbles of the Future?

Exosomes are small cup-shaped extracellular vesicles (50–150 nm in size) released from the plasma membrane by almost all cells, including cancer cells (Figure 1) (24). They play an important role in intercellular communication, tumorigenesis and cancer metastasis (97–100). Structurally, they are enveloped by a lipid bilayer membrane with tissue-specific content instead of cellular organelles, such as pathogenic mRNA, microRNA, DNA fragments, and proteins (101, 102). After release, exosomes are stable in the extracellular environment or enter the circulation and can be taken up by neighbor or distant cells (103, 104). Exosomes allow the exchange of material and information between cells, altering gene expression or mediating RNA silencing (105).

Considering their extensive distribution and functions, exosomes are ideal candidates to find circulating biomarkers for PC detection and management (67, 104, 106). Moreover, exosomes have some advantages over other biomarkers (106, 107). First, they are widely distributed in nearly all body fluids, including serum, and are relatively stable when stored for long term at −80°C. Second, cancer cells secrete more exosomes than normal healthy cells. One of the reasons for this can be the acidic conditions of the tumor microenvironment that enhances the release of exosomes (108–110). Third, exosomes contain a combination of proteins, DNA, coding and non-coding RNAs, and lipids that can be used as a natural panel of biomarkers for simultaneous evaluation. Fourth, PC-derived exosomes enter the circulation at an early stage of cancer development and are related to metastasis. In fact, on the contrary of some other cancer markers that are released into the blood only after necrosis-related cell death occurs, a phenomenon usually associated with advanced stage disease with large volume tumor mass, cell-secreted exosomal nucleic acids (DNA fragments, mRNA, miRNAs, and others) are released in circulation during the initial phases of the tumorigenesis process (66, 91, 107). This aspect is critical for the early detection of PC because PC cells are able to metastasize at an early stage, with great impact on prognosis.

Recent studies have implicated PC-derived exosomes in the early development of PC (103) and showed that they contribute for establishing a premetastatic niche in the liver (98, 111) and that they can promote tumor formation and proliferation (112). In relation to exosomes' content, a main focus of research has been on RNA and microRNA profiling, in part due to the established utility of a variety of some of these molecules in cancer screening and also due to the relative ease feasibility of its amplification (99, 113–118). By turn, in order to determine their cellular origin, exosomal proteomic profiling is being an important focus of research in recent years (114).

Besides their possible role in diagnosis, the study of exosomal function has contributed to the improvement of the comprehension of the microenvironment related to PC and progression of the disease. The ultimate aim of understanding the way exosomes can influence tumor initiation, proliferation and metastasis is improving the knowledge on PC pathophysiology and patient prognosis. Moreover, its role in the development of some paraneoplastic conditions, such as diabetes *mellitus* and cachexia, and in the resistance to chemotherapy, can provide insights for future therapeutic targets (119–125).

The extraction of exosomes from body fluids has been described using various methods and technologies. Commonly used methods for isolation are ultracentrifugation, precipitation, size, immunoaffinity, and microfluidics (126).

Strong efforts have focused on developing sensitive diagnostics tools improving early detection of PC via identifying pancreatic cancer-associated exosomal markers (67, 104, 127). The first case–control study on PC exosomes was conducted by Que et al. (128), where four exosomal microRNAs were evaluated as candidates. In this study, a moderate discrimination of cases from controls was seen with miR-21 and miR-17-5p. In 2015, in a study involving patients with PC, chronic pancreatitis and controls, a higher expression of exosomal miR-10b was shown only in cancer patients (129). Although this cohort was a small one, the strength of this study was the use of new technology of label-free nanoplasmonic-based short non-coding RNA sensing. In the same year, a more extended cohort including PC patients, patients with chronic pancreatitis, individuals with benign pancreatic neoplasms and controls was evaluated by Madhavan et al. (130). In this well conducted study, a combination of four miRNAs (miR-1246, miR-4644, miR-3976, and miR-4306) and five proteins (CD44v6, Tspan8, EpCAM, MET and CD104) in circulating exosomes was able to distinguish PC patients from non-cases ones, with a sensitivity of 1.0 (CI: 0.95-1) and a specificity of 0.80 (CI: 0.67-0.90).

In the past years, the number of exosomal miRNAs studied in this context was considerable. Examples of these molecules are miR-21, miR-17-5p, miR-155, miR-34, miR- 196a, miR-181a, miR-181b, miR-138-5p, miR-494, miR- 542-3p, miR-31, and miR-205, that have been implicated for several studies to have the capacity, when upregulated, to promote cellular proliferation and angiogenesis, metastasis and disease progression and even chemo-resistance in PC patients (113, 128, 131–138).

These studies emphasize the importance of exosomal miRNAs not only as diagnostic and prognostic biomarkers, but also open a field of investigation of its use as potential targets for treatment PC.

Recently, Melo et al. showed that glypican-1 (GPC1), a membrane anchored protein, in circulating exosomes may distinguish with 100% specificity patients with PC or precancerous pancreatic lesions from patients with benign pancreatic diseases (139). Melo et al. reported that GPC1 expression patterns in exosomes secreted by PC could be utilized to identify subjects with PC early and offer considerable insights into the disease progress and tumor load. A comparison of exosomes from PC and control cell lines indicated that the exosomes from cancer exhibited enhanced levels of GPC1. In serum specimens from subjects with PC (*n* = 190), a significantly larger amount of GPC1+ circulating exosomes was present compared with normal controls (*n* = 100). Interestingly, direct analysis of GPC1 in serum itself revealed lower sensitivity and specificity than measurement of GPC1 in purified serum exosomes. Furthermore, GPC1+ exosomes were also confirmed to contain identical KRAS mutations, which frequently are present in PC and precancerous lesions and have been considered a fundamental mutation (139). Moreover, higher levels of GPC1 positive circulating exosomes were seen in both PC and PC precursor lesions, such as IPMNs, when compared to other benign diseases of the pancreas and healthy controls, with a perfect area under the receiver-operating characteristic curve (AUC) of 1.0. The authors concluded that given the high sensitivity and specificity of exosomal GPC1 in differentiating PC, independently of its stage, from non-PC controls, this biomarker could have a promising role in PC early detection. Not surprisingly, when compared to the tumor marker CA 19-9 (AUC: ranging between 0.69 and 0.79), exosomal GPC1 was found to be significantly superior (*p* < 0.001) (139). Additionally, using a genetically engineered mouse that progressively developed into PC, the identification of GPC1+ exosomes exhibited positive results prior to pancreatic lesions being detectable by MRI. Tumor burden was associated positively with levels of GPC1+ circulating exosomes. In most subjects, the exosome levels reduced following the removal of the solid tumor. In this manner, circulating exosomal GPC1 may be seen as a prognostic indicator too, reflecting tumor load and monitoring disease progression and patients' survival. Moreover, evolving research will address if this biomarker can play a role in the treatment of PC as a potential pharmacological target (139). Many others since than have reported findings related to GPC1 detection in exosomes, not only in PC but also breast and colorectal cancer cases (140–148). In any event, in the future, further independent confirmation of exosomal GPC1 performance is needed, assessing its role as a more reliable marker in predicting diagnosis and prognosis of PC when compared to CA19-9, but also, and as it seems to be overexpressed in some precursor lesions such as IPMNs, its potential role in individualizing the management of such conditions (149).

Also interestingly, a paper from Hoshino et al. (98) has described that integrin αvβ5-expressing PC exosomes can determine liver metastasis and that targeting that molecule reduced exosome absorption by resident cells and inhibited liver involvement. The levels of integrin αvβ5 were noticeably enhanced in exosomes isolated from PC individuals with liver metastasis compared with those with no distant metastasis. Together, these findings have raised the possibility that GPC1+ and integrin αvβ5-expressing circulating exosomes may be used as indicators of PC progression and liver metastization.

Taking in account the excellent biodistribution and biocompatibility of exosomes, the idea of its utilization as vehicles for drug, genes or nucleic acids delivery has gained increased acceptance for continuous research in the field of PC treatment (124, 150, 151), especially when considering the fact that they can be particularly targeted to specific cell types by engineered exosomes-producer cells (124, 150, 152). This is important especially when dealing with a tumor with such difficulties in therapy delivery due to the intrinsic resistance of its microenvironment and its dense stroma (153).

## Conclusion and New Pathways of Investigation

Given the timeframe from pancreatic tumor initiation to invasion and metastatic capacity, there is a large window of opportunity for early management of this lethal disease. An ideal diagnostic method for PC should definitively distinguish malignant lesions from benign ones and detect early-stage disease and preneoplastic conditions, such as PanINs and cystic mucinous lesions with risk of progression.

There are many challenges in the early detection of PC, including its asymptomatic nature, the lack of imaging exams able to detect minimal lesions and the absence so far of sensitive and specific molecules in body fluids. Recently described circulating biomarkers associated with PC initiation and progression, easily detectable in blood, followed by confirmative diagnosis based on imaging and pathologic results might be the future ideal strategy for screening and diagnosing PC.

A number of circulating biomarkers have been widely studied, but validation for routine clinical use is still needed. The lack of sufficient samples from non-invasive precursor lesions and early-stage PC must be addressed, and animal models are important tools for research. In fact, extensive understanding of the fundamentals of PC development and the nature of precursor lesions is crucial prerequisite toward discovering and applying novel biomarkers.

Exosomes have been proving to be reliable candidates as PC biomarkers, as its contents, namely DNA, RNASs, proteins, lipids, and metabolites are largely derived from tumor cells. In this way, tracking these molecules will conduct to the knowledge of cell-type of origin, and also importantly the specificity for pre-metastatic niches formation and distant colonization.

Its potential utilization as biomarkers, besides the ultimate goal of early detection of PC, can also play an important role in monitoring disease progression.

Moreover, considering its excellent biodistribution, biocompatibility and cell-specific nature, exosomes can be used, in the future, as drug delivery vehicles.

The majority of studies addressing exosomes as biomarkers have been based on patients with established diagnosis of PC. However, and having in mind that when facing this dismal disease, the earlier the diagnosis the better the chance for cure, it would be of great interest to access the performance of these biomarkers in the context of high-risk individuals screening.

Continuous investigation is needed to ascertain the potential of these biomarkers, so that, hopefully, in the near future, they will provide the opportunity for early diagnosis, treatment intervention and increasing survival of PC patients.

## Author Contributions

PM-R did the bibliographic search and wrote the initial manuscript. GM and SM critically revised the manuscript and actively contributed to it.

### Conflict of Interest Statement

SM has ownership interests (patents). The remaining authors declare that the research was conducted in the absence of any commercial or financial relationships that could be construed as a potential conflict of interest.
